# Supplementary material for: Dynamic Interrogation of Stochastic Transcriptome Trajectories Using Disease Associated Genes Reveals Distinct Origins of Neurological and Psychiatric Disorders
Source: Front Neurosci. 2022 Jun 2;16:884707. doi: 10.3389/fnins.2022.884707 (PMC9201694; doi:10.3389/fnins.2022.884707)
Supplement: Supplementary file 1 [file Data_Sheet_1.pdf]

Supplementary Material for **Dynamic Interrogation of Stochastic Transcriptome Trajectories Using Disease Associated Genes Reveals Distinct Origins of Neurological and Neuropsychiatric Disorders**

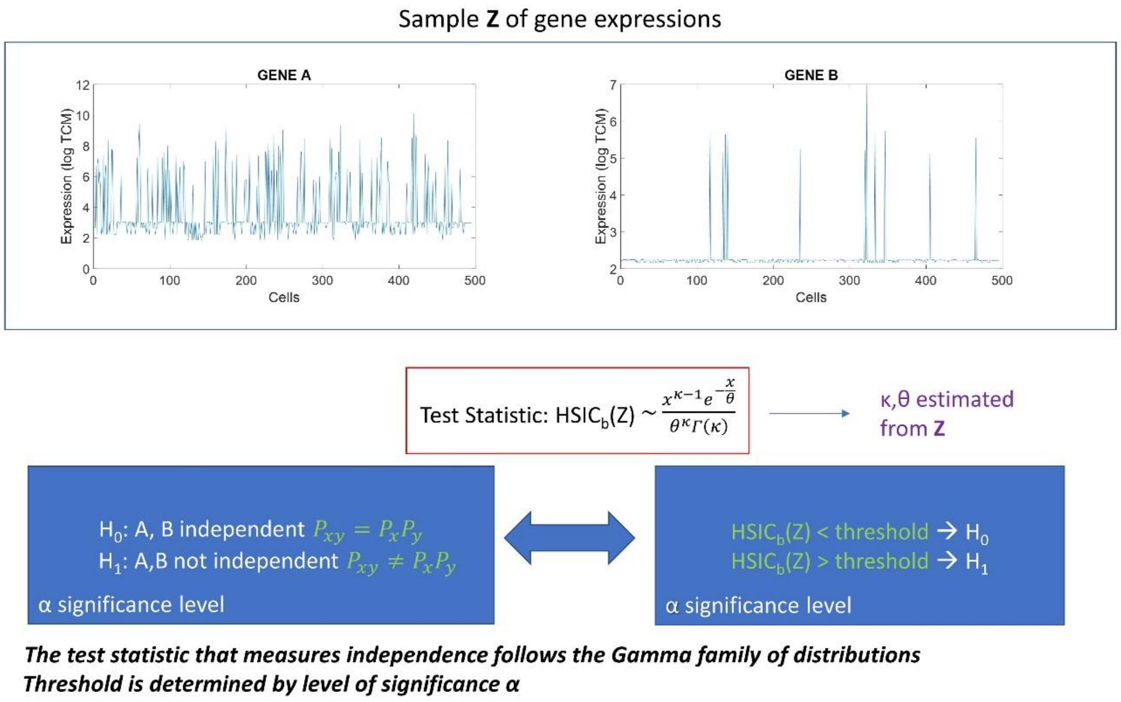

Figure 0. Schematics of analyses for level 2 of inquiry in the main text methods section for the Kernel Statistical Test of Independence by Gretton et al. [17] applied on the gene expressions of a pair of genes in the hESCs.

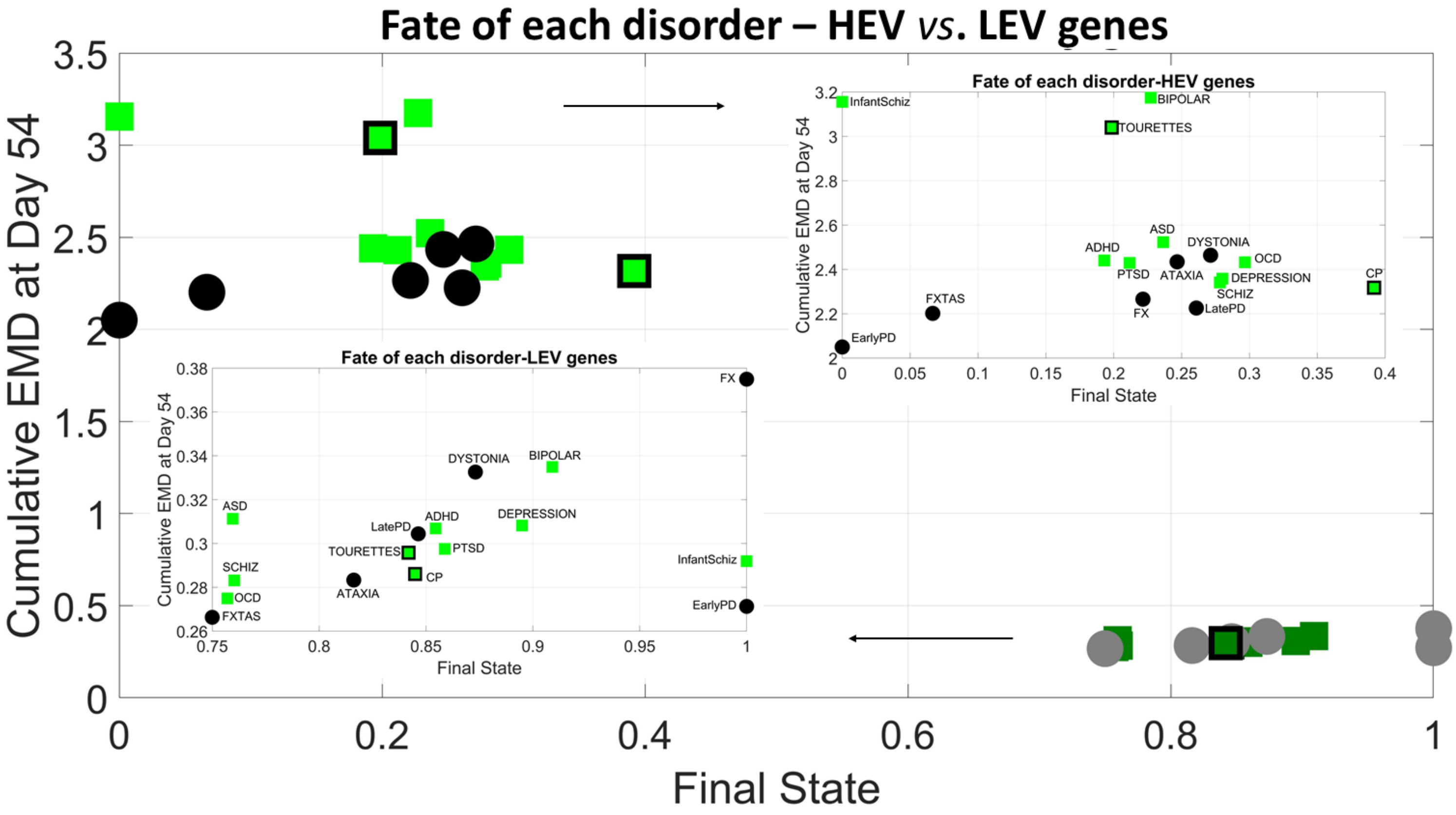

Figure 1. This figure expands on Figure 5 of the main text highlighting the differences in cumulative EMD at date 54 (fate) and the final state of the gene's dependency index. Insets show the individual disorders in each cluster. Bright green and black on the upper left corner are HEV genes and grey with darker green on the lower right corner of the parameter plane are LEV genes. Squares are psychiatric and circles are neurological disorders. Those green squares with a black edge are disorders which can be diagnosed with the DSM criteria as psychiatric, but are also considered neurological (*e.g.,* Cerebral Palsy and Tourette's, which share genes in each set)

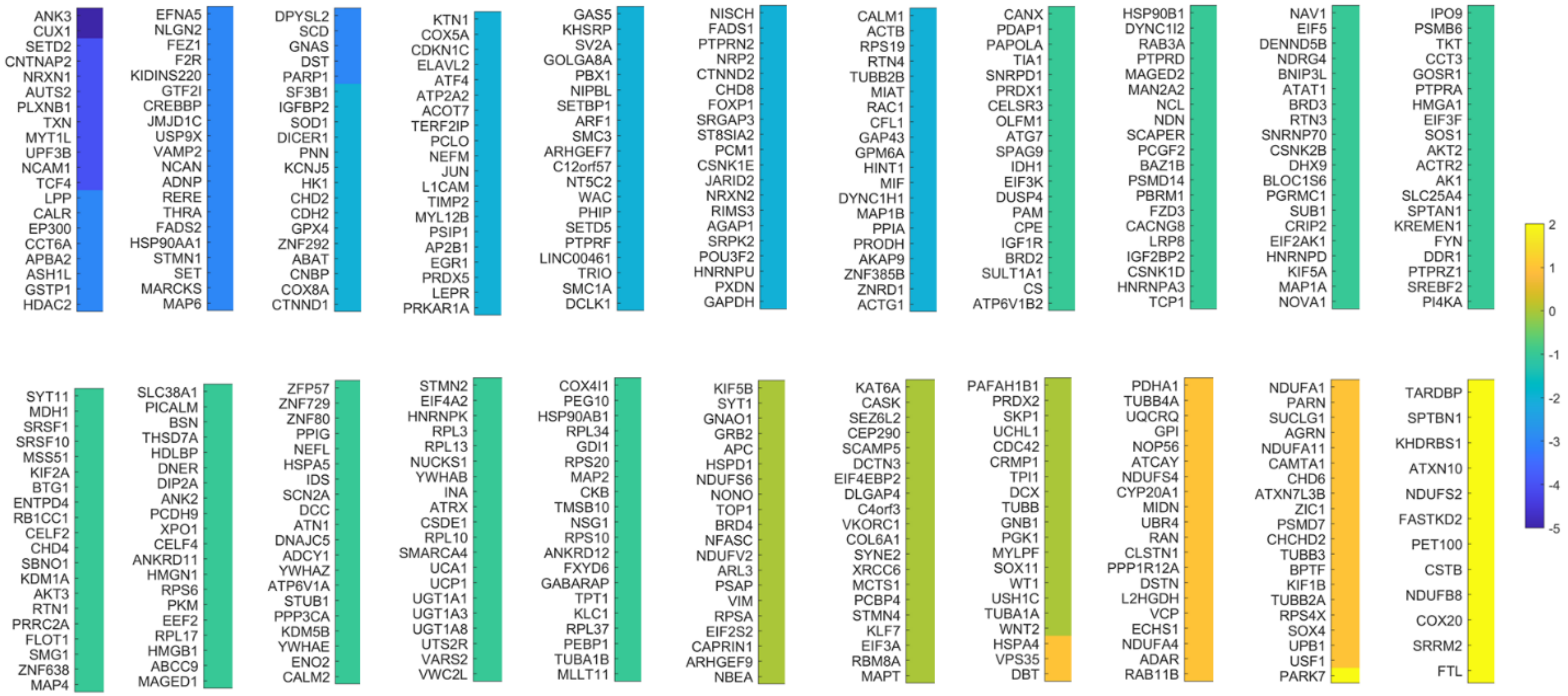

Figure 2. Colormap depicting genes sorted by disease index ratio (Equation 14) weighing the balance between HEV and LEV genes in neurological *vs*. psychiatric disorders. HEV genes are plotted in the order of the scalar quantity (log of the ratio) whereby the higher the value (yellow), the higher the weight in neurological disorders. This figure expands from Figure 10 in the main text.

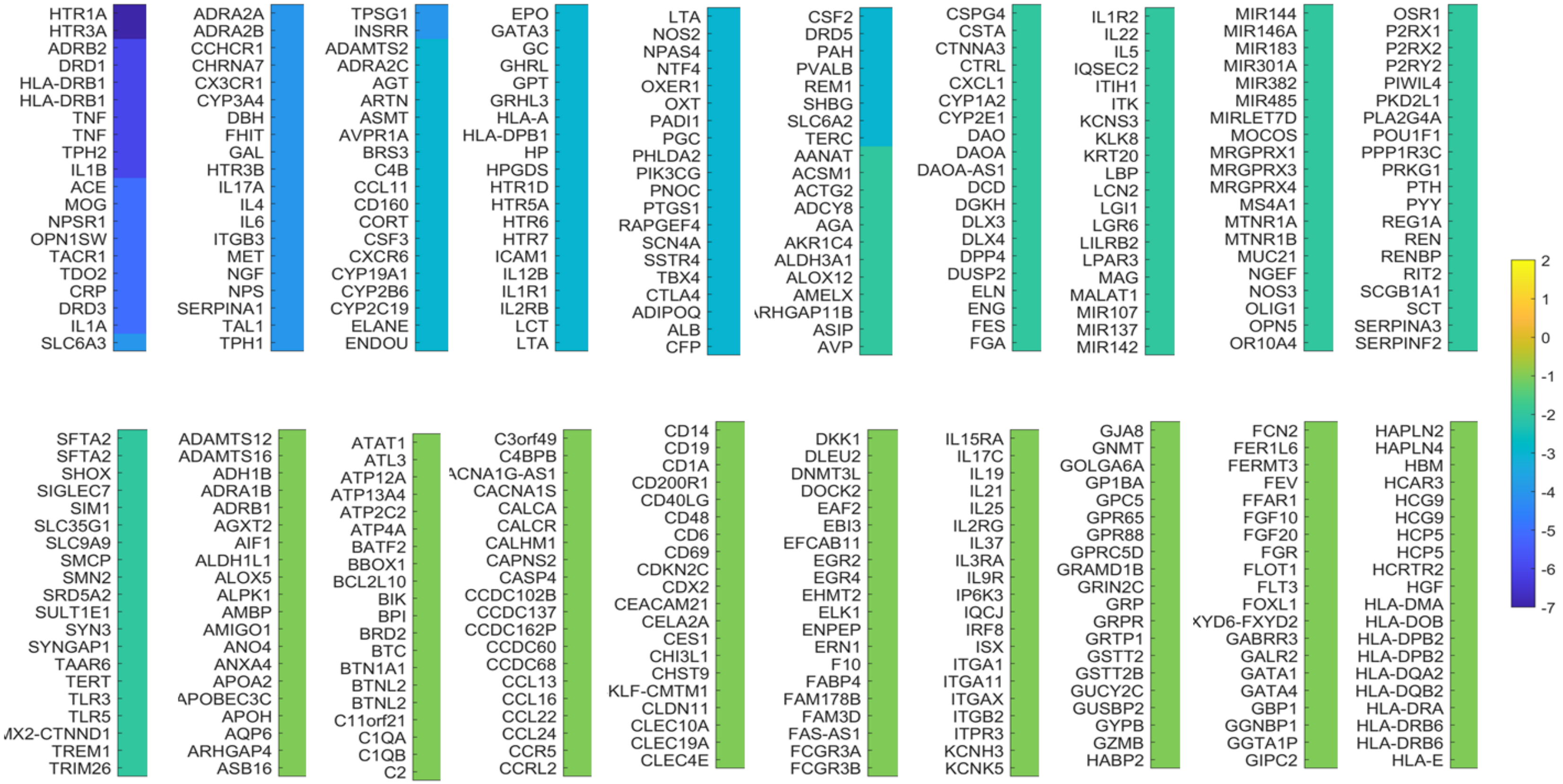

Figure 3. LEV genes color coded as in Figure 2, based on the log of the disease index ratio. This figure expands on Figures 11-12 in the main text.

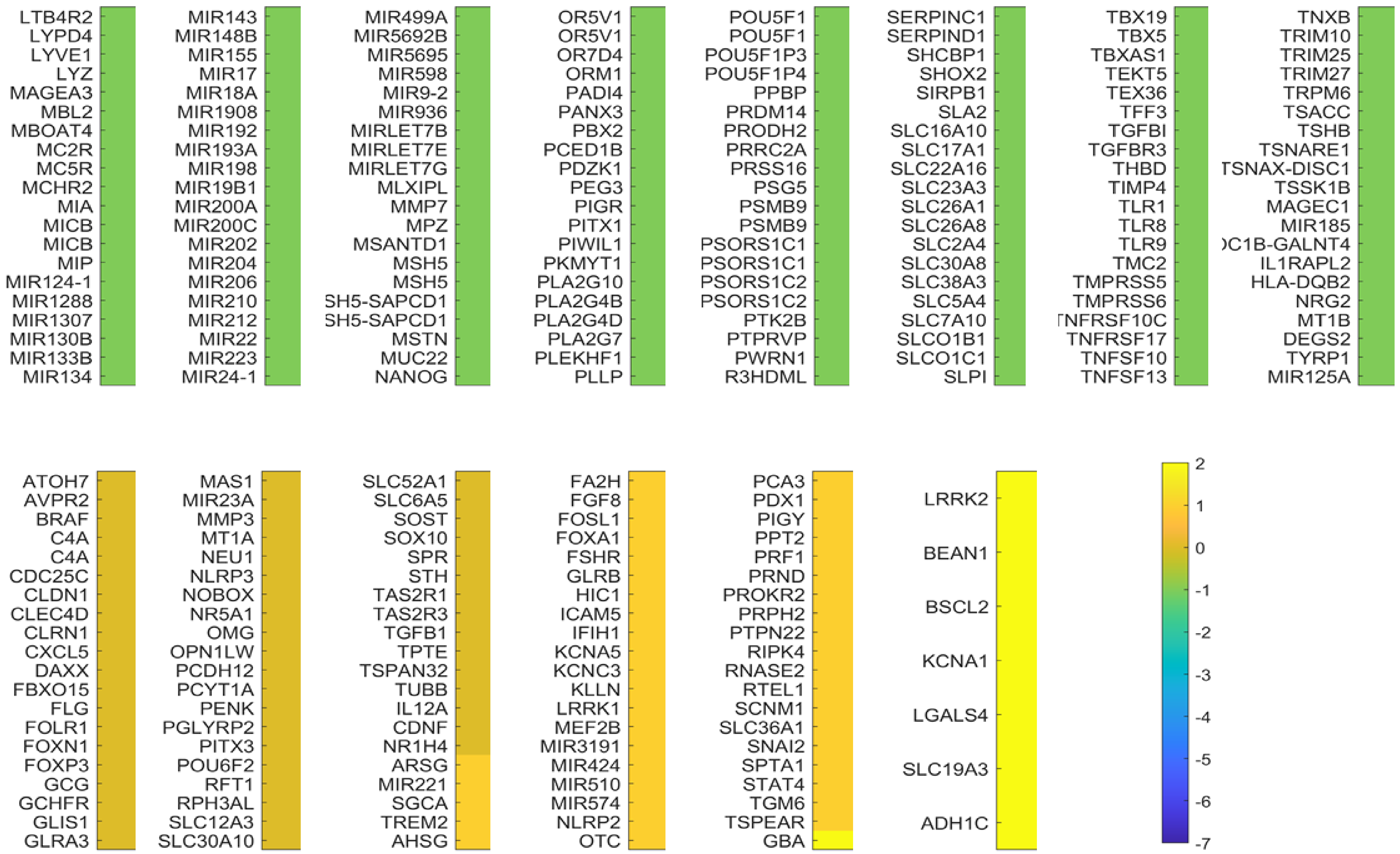

Figure 4. LEV genes (continued) color coded as in Figure 2-3, based on the log of the disease index ratio.

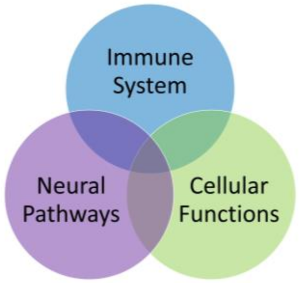

Figure 5. Venn diagram highlighting the three main clusters of genes identified by OMIM according to their function. Many genes play major roles at the interface between the immune system and the nervous system (See table in Supplementary Material). When considering psychiatric disorders alone or neurological disorders alone, very few high degree genes were found in neurological disorders alone. All three types of genes were found in genes common to both neurological **and** psychiatric disorders. For example, ELAVL4 of degree 165 was found in Late PD and Schizophrenia but it is also reported in paraneoplastic neurological disorders, and in autoimmune neuronal degeneration.

Table 1. Catalogue of High Expression Variability (HEV) genes and Low Expression Variability (LEV) genes. See Figures 8 and 9 in the main text corresponding to the HEV (red) and LEV (blue) genes evolving networks. Each row corresponds to the disorders in the column of Neurological and Psychiatric or Neurological only entries, each row corresponds to the gene in the Genes column entry, with network node degree in the Degree column and Day column

Neurological and Psychiatric	Genes	Degree	Day
Ataxia, Schiz, ADHD,	YWHAG	7	12
FXTAS, SCHIZ, Depression	YWHAZ	78	
Ataxia, Depression	ABCC8	19	
Ataxia, FX, Schiz, ADHD, Depression	APP	20	19
Ataxia, Schiz	PGK1	45	
Late PD,Schiz	ELAVL4	165	
Late PD, Ataxia, Depression, Tourette’s	NLRP3	7	
Dystonia, ADHD, ASD	CDKL5	20	
Early PD, Late PD, Dystonia, Schiz, ADHD, PTSD, Depression	SLC18A2	20	
Dystonia, ADHD, ASD	CDKL5	3	40
Late PD, Schiz, Depression	IL2RA	3	
Late PD, FXTAS, Depression	SGCA	3	
Late PD, Schiz, ADHD, Depression, Tourette’s	SLC6A2	3	
Late PD, Schiz, ADHD, PTSD, OCD	TAL1	3	
Late PD, Ataxia, Schiz, Depression	TGFB1	3	

Dystonia, Schiz<br>Ataxia, Depression<br>Ataxia, Schiz<br>Late PD, ASD<br>Ataxia, Schiz<br>Late PD, Schiz	GCHFR<br>NR5A1<br>PCYT1A<br>BRAF<br>RFT1<br>STH Chr17ctg5 hap	4<br>4<br>4<br>7<br>13<br>26	
Late PD, Schiz<br>Late PD, Schiz, ADHD, PTSD, Depression, OCD, Tourette's<br>Ataxia, Schiz<br>Early PD, Schiz<br>Late PD, Schiz, ADHD, Depression	WNT2<br>IL1B<br>IL12A<br>CDNF<br>MSMB	16<br>6<br>7<br>33<br>342	54
Neurological Only			
None			12
None			19
Dystonia, Ataxia	KCNA1	6	40
Late PD, FXTAS	SGCA	3	54
Psychiatric Only			
Schiz, ADHD, Depression	SCD	11	12
Infantile Schiz, ASD<br>Schiz, PTSD, Depression<br>Schiz, Depression<br>Schiz, Depression<br>Schiz, ASD, PTSD<br>Schiz, ADHD, Depression<br>ADHD, Depression<br>Schiz, ASD<br>OCD, Tourette's<br>Schiz, Depression<br>PTSD, Depression<br>Schiz, Bipolar, Tourette's<br>Schiz, Depression	PXDN<br>STMN1<br>DENND5B<br>CDKN1C<br>SET<br>ADRA2C<br>MIRLET7D<br>CNTNAP3<br>POU1F1<br>CRHBP<br>PKD2L<br>ILRL1<br>MALAT1	5<br>7<br>13<br>78<br>156<br>4<br>6<br>6<br>8<br>11<br>12<br>20<br>153	19
Schiz, ADHD<br>Schiz, Depression<br>Schiz, ADHD, Depression<br>Schiz, PTSD<br>Schiz, ASD<br>Schiz, Bipolar, Tourette's<br>Schiz, ADHD, PTSD, Depression, OCD	PPIA<br>CRHBP<br>ADAMTS2<br>DUSP2<br>HS3ST5<br>IL1R1<br>NPSR1	5<br>2<br>3<br>3<br>3<br>3<br>3	40

Schiz, ADHD<br>ADHD, Depression<br>Schiz, Depression<br>ADHD, Depression<br>ADHD, Depression<br>Schiz, ADHD<br>Schiz, Bipolar, Depression<br>Schiz, Depression<br>Schiz, ASD, Depression<br>Schiz, Depression<br>Schiz, OCD, Tourette's<br>ASD, OCD<br>ADHD, ASD<br>Schiz, Depression, OCD<br>Schiz, ADHD<br>Schiz, Depression, OCD, Tourette's<br>Schiz, ADHD<br>Schiz, Depression	PRKG1<br>GALR1<br>LIF<br>SIM1<br>CHRNA6<br>DGKH<br>NPAS4<br>TREM1<br>RAPGEF4<br>KLK8<br>LCT<br>CDH9<br>IQSEC2<br>PGC<br>PIWIL4<br>HTR3B<br>CNTNAP3<br>MALAT1	3<br>4<br>4<br>4<br>5<br>5<br>5<br>5<br>6<br>7<br>7<br>9<br>9<br>9<br>10<br>11<br>20<br>179	
Schiz, ADHD, ASD, Depression<br>Schiz, ADHD<br>ADHD, OCD	TCF4<br>CD40<br>GJB2	19<br>15<br>28	54

Table 2. Glossary of terms

Term	Definition
Hight Expression Variability (HEV) gene	A gene belonging to the class of genes with a high variance of expression in the cells
Low Expression Variability (LEV) gene	A gene belonging to the class of genes with a low variance of expression in the cells
Hub gene	A gene with a high node degree in the Chow Liu graphical model
Independent genes	Two genes with expression profiles that are statistically independent
Conditionally Independent genes	Two genes, from a set of genes, with expression profiles that are statistically independent given the behavior of all other genes from that set
Synchronicity	Refers to the behavior of two genes that follow similar expression patterns through time. Both genes are over-expressed or inactive at some point in time

Table 3. Extended HEV (red) and LEV (blue) information compiled from Online Mendelian Inheritance in Man (OMIM) database.

Neurological and Psychiatric	Phenotype
Ataxia, Schiz, ADHD, FXTAS, Ataxia, Depression	YWHAG. Developmental and epileptic encephalopathy-56 (DEE56) is a neurodevelopmental disorder characterized by early-onset seizures in most patients, followed by impaired intellectual development, variable behavioral abnormalities, and sometimes additional neurologic features, such as ataxia (summary by Guella et al., 2017) Guella, I., McKenzie, M. B., Evans, D. M., Buerki, S. E., Toyota, E. B., Van Allen, M. I., Epilepsy Genomics Study, Suri, M., Elmslie, F., Deciphering Developmental Disorders Study, Simon, M. E. H., van Gassen, K. L. I., Heron, D., Keren, B., Nava, C., Connolly, M. B., Demos, M., Farrer, M. J. **De novo mutations in YWHAG cause early-onset epilepsy.** Am. J. Hum. Genet. 101: 300-310, 2017. [PubMed: 28777935]<br><br>YWHAZ Popov et al. (2019) described a neurodevelopmental disorder characterized by global developmental delay apparent from infancy. Affected individuals had impaired intellectual development and poor or absent speech, as well as behavioral abnormalities. Most patients had significant facial dysmorphism, including coarse features, frontal bossing, and abnormal eye shape. Additional features were highly variable and included seizures, short stature, feeding difficulties, and skin abnormalities. For discussion of a possible association between this neurodevelopmental disorder and mutation in the YWHAZ Popov, I. K., Hiatt, S. M., whalen, S., Keren, B., Ruivenkamp, C., van Haeringen, A., Chen, M.-J., Cooper, G. M., Korf, B. R., Chang, C. **A YWHAZ variant associated with cardiofaciocutaneous syndrome activates the RAF-ERK pathway.** Front. Physiol. 10: 388, 2019. Note: Electronic Article. [PubMed: 31024343, images, related citations] [Full Text]<br><br>ABCC8 permanent neonatal diabetes neonatal diabetes, Babenko et al. (2006) screened the ABCC8 gene in 34 who did not have alterations in chromosome 6q or mutations in the KCNJ11 or GCK (138079) genes. In 2 patients with permanent neonatal diabetes, they identified heterozygosity for a mutation
Ataxia, FX, Schiz, ADHD, Depression<br>Ataxia, Schiz<br>Late PD,Schiz<br>Late PD, Ataxia, Depression, Tourette's<br>Dystonia, ADHD, ASD<br>Early PD, Late PD, Dystonia, Schiz, ADHD, PTSD, Depression	APP 21q21.3 Alzheimer disease 1, familial<br>PGK1 Xq21.1 Phosphoglycerate kinase 1 deficiency which catalyzes the reversible conversion of 1,3-diphosphoglycerate to 3-phosphoglycerate during glycolysis, generating one molecule of ATP<br>ELAVL4 The paraneoplastic neurologic disorders (PND) are a rare group of neurologic syndromes that arise when an immune response to systemic tumors expressing neuronal proteins ('onconeural antigens') develops into an autoimmune neuronal degeneration. PD<br>NLRP3 CINCA syndrome, also known as 'neonatal onset multisystem inflammatory disease,' or NOMID, is a rare congenital inflammatory disorder characterized by a triad of neonatal onset of cutaneous symptoms, chronic meningitis, and joint manifestations with recurrent fever and inflammation (Prieur and Griscelli)

	CDKL5 deficiency disorder is characterized by seizures that begin in infancy, followed by significant delays in many aspects of development. Seizures in CDKL5 deficiency disorder usually begin within the first 3 months of life, and can appear as early as the first week after birth<br>SLC18A2 evidence that infantile parkinsonism-dystonia-2 (PKDYS2) is caused by homozygous mutation on chromosome 10q25
Dystonia, ADHD, ASD, Late PD, Schiz, Depression, FXTAS, Tourette's, PTSD, OCD, Ataxia, Dystonia, Ataxia	IL2RA Immunodeficiency 41 with lymphoproliferation and autoimmunity, Diabetes, mellitus, insulin-dependent, susceptibility to<br>SGCA Muscular dystrophy, limb-girdle, autosomal recessive 3 17q21.33 autosomal recessive limb-girdle muscular dystrophy-3 (LGMDR3) is caused by homozygous or compound heterozygous mutation in the alpha-sarcoglycan gene (SGCA)<br>SLC6A2 Orthostatic intolerance 16q12.2 encodes a norepinephrine (noradrenaline) transporter, which is responsible for reuptake of norepinephrine into presynaptic nerve terminals and is a regulator of norepinephrine homeostasis (Kim et al., 2006) Kim, C.-H., Hahn, M. K., Joung, Y., Anderson, S. L., Steele, A. H., Mazei-Robinson, M. S., Gizer, I., Teicher, M. H., Cohen, B. M., Robertson, D., Waldman, I. D., Blakely, R. D., Kim, K.-S. **A polymorphism in the norepinephrine transporter gene alters promoter activity and is associated with attention-deficit hyperactivity disorder.** Proc. Nat. Acad. Sci. 103: 19164-19169, 2006. [PubMed: 17146058]<br><br>TAL1 Autoimmune thyroid disease, susceptibility 8q24.22<br>TGFB1 19q13.2 Camurati-Engelmann disease, Inflammatory bowel disease, immunodeficiency, and encephalopathy Cystic fibrosis lung disease TGFB is a multifunctional peptide that controls proliferation, differentiation, and other functions in many cell types. TGFB acts synergistically with TGFA (190170) in inducing transformation. It also acts as a negative autocrine growth factor. Dysregulation of TGFB activation and signaling may result in apoptosis. Many cells synthesize TGFB and almost all of them have specific receptors for this peptide. TGFB1, TGFB2 (190220), and TGFB3 (190230) all function through the same receptor signaling systems<br>GCHFR 15q15.1 protein in rat that bound to GTP cyclohydrolase I (600225) and exhibited tetrahydrobiopterin-dependent inhibition of that enzyme. They found that this regulatory protein, which they termed GFRP, consists of a homodimer of 9.5-kD subunits and has a molecular mass of 20 kD. Milstien et al. (1996) used peptide sequences to clone the corresponding rat cDNA which encodes an 84-amino acid polypeptide. Northern blot analysis of rat tissues revealed that a 0.8-kb GFRP transcript was expressed at relatively high levels in liver and kidney and at somewhat lower levels in testis, heart, brain, and lung. Milstien et al. (1996) suggested that GFRP may play a role in regulating phenylalanine metabolism in the liver and in the production of biogenic amine neurotransmitters and nitric oxide<br><br>NR5A1 9q33.3 46, XX sex reversal 46XY sex reversal 3<br><br>PCYT1A 3q29 Spondylometaphyseal dysplasia-cone-rod dystrophy syndrome is characterised by the association of spondylometaphyseal dysplasia (marked by platyspondyly, shortening of the tubular bones and progressive metaphyseal irregularity and cupping), with postnatal growth retardation and progressive visual impairment due to cone

	BRAF Noonan syndrome is a condition that some babies are born with. It causes changes in the face and chest, usually includes heart problems, and slightly raises a child's risk of blood cancer (leukemia). Noonan syndrome is a common condition, affecting 1 in 1,000–2,500 babies. Delayed puberty<br>Down-slanting or wide-set eyes, hearing loss (varies), Low-set or abnormally shaped ears, Mild intellectual disability (only in about 25% of cases), Sagging eyelids (ptosis), Short stature, small genitalia, undescended testicles, unusual chest shape (most often a sunken chest called pectus excavatum). Webbed and short-appearing neck<br><br>RFT1 3p21.1 Congenital disorder of glycosylation N-glycosylation of proteins follows a highly conserved pathway that begins with the synthesis of a Man(5)GlcNAc(2)-dolichylpyrophosphate (PP-Dol) intermediate on the cytoplasmic side of the endoplasmic reticulum (ER) membrane followed by the translocation of Man(5)GlcNAc (2)-PP-Dol to the luminal side of the ER membrane. RFT1 is the flippase enzyme that catalyzes this translocation (Helenius et al., 2002)<br><br>STH Chr17ctg5 hap Alzheimer Conrad, C., Vianna, C., Freeman, M., Davies, P. A polymorphic gene nested within an intron of the tau gene: implications for Alzheimer's disease. Proc. Nat. Acad. Sci. 99: 7751-7756, 2002. [PubMed: 12032355]<br><br>Verpillat, P., Ricard, S., Hannequin, D., Dubois, B., Bou, J., Camuzat, A., Pradier, L., Frebourg, T., Brice, A., Clerget-Darpoux, F., Deleuze, J.-F., Campion, D., the French Study Group on Alzheimer's Disease and Frontotemporal Dementia. Is the saitohin gene involved in neurodegenerative diseases? Ann. Neurol. 52: 829-832, 2002. [PubMed: 12447938]
Late PD, Schiz, ADHD, PTSD, Depression, OCD, Tourette's<br>Ataxia, Early PD	WNT2 1p13.2 neonatal-onset chronic diarrhea O'Connell, A. E., Zhou, F., Shah, M. S., Murphy, Q., Rickner, H., Kelsen, J., Boyle, J., Doyle, J. J., Gangwani, B., Thiagarajah, J. R., Kamin, D. S., Goldsmith, J. D., Richmond, C., Breault, D. T., Agrawal, P. B. Neonatal-onset chronic diarrhea caused by homozygous nonsense WNT2B mutations. Am. J. Hum. Genet. 103: 131-137, 2018. [PubMed: 29909964, related citations] [Full Text] Ober, E. A., Verkade, H., Field, H. A., Stainier, D. Y. R. Mesodermal Wnt2b signalling positively regulates liver specification. Nature 442: 688-691, 2006. [PubMed: 16799568, related citations] [Full Text]<br><br>IL1B 2q14.1 Gastric cancer risk after H. pylori infection Interleukin-1, produced mainly by blood monocytes, mediates the panoply of host reactions collectively known as acute phase response. It is identical to endogenous pyrogen. The multiple biologic activities that define IL1 are properties of a 15- to 18-kD protein that is derived from a 30- to 35-kD precursor<br>IL12A 3q25.33 INTERLEUKIN 12A<br><br>CDNF 10p13 CONSERVED DOPAMINE NEUROTROPHIC FACTOR<br>MSMB 10q11.22 Prostate cancer, hereditary
Neurological Only	
Dystonia, Ataxia	KCNA1 12p13.32 Episodic ataxia/myokymia syndrome Potassium channels represent the most complex class of voltage-gated ion channels from both functional and structural standpoints. Present in all eukaryotic cells, their diverse functions include maintaining membrane potential, regulating cell volume, and modulating electrical excitability in neurons. The delayed rectifier function of potassium channels allows nerve cells to efficiently repolarize following an action potential
Late PD, FXTAS	SGCA 17q21.33 Muscular dystrophy, limb-girdle, autosomal recessive 3
